# ZnO-PLLA/PLLA Preparation and Application in Air Filtration by Electrospinning Technology

**DOI:** 10.3390/polym15081906

**Published:** 2023-04-16

**Authors:** Xinxin Liu, Dengbang Jiang, Yuyue Qin, Zhihong Zhang, Mingwei Yuan

**Affiliations:** 1 Green Preparation Technology of Biobased Materials National & Local Joint Engineering Research Center, Yunnan Minzu University, Kunming 650500, China; 2 Institute of Agriculture and Food Engineering, Kunming University of Science and Technology, Kunming 650550, China; 3 School of Food and Biological Engineering, Jiangsu University, Zhenjiang 212013, China

**Keywords:** nano ZnO, electrospinning, polylactide, air filtration, orthogonal experiments

## Abstract

With the increasing environmental pollution caused by disposable masks, it is crucial to develop new degradable filtration materials for medical masks. ZnO-PLLA/PLLA (L-lactide) copolymers prepared from nano ZnO and L-lactide were used to prepare fiber films for air filtration by electrospinning technology. Structural characterization of ZnO-PLLA by H-NMR, XPS, and XRD demonstrated that ZnO was successfully grafted onto PLLA. An L_9_(4^3^) standard orthogonal array was employed to evaluate the effects of the ZnO-PLLA concentration, ZnO-PLLA/PLLA content, DCM(dichloromethane) to DMF(N,N-dimethylformamide) ratio, and spinning time on the air filtration capacity of ZnO-PLLA/PLLA nanofiber films. It is noteworthy that the introduction of ZnO is important for the enhancement of the quality factor (QF). The optimal group obtained was sample No. 7, where the QF was 0.1403 Pa^−1^, the particle filtration efficiency (PFE) was 98.3%, the bacteria filtration efficiency (BFE) was 98.42%, and the airflow resistance (Δp) was 29.2 Pa. Therefore, the as-prepared ZnO-PLLA/PLLA film has potential for the development of degradable masks.

## 1. Introduction

With the long-term impact of COVID-19, influenza, and other respiratory diseases, disposable masks are being used and discarded at a rate of 130 billion per month worldwide [1,2]. Traditional disposable masks are mainly made of polyester, polyethylene, polypropylene, and other raw materials [3,4]. However, the natural degradation process of these materials is slow and poses a huge impact on the environment. The adoption of green, biodegradable materials to replace traditional polymer materials is an effective means to further protect the ecological environment [5,6].

What is needed in the development of medical masks is not only new ideas and new technological breakthroughs, but also the introduction of new materials. Compared with petroleum-based polymers, polylactic acid (PLA) has received much attention as a biodegradable hydrophobic polymer [7,8,9]. PLA synthetic raw materials are widely available and inexpensive and have been used in the packaging [10], medical [11], and textile fields [12]. Therefore, they have become one of the best alternative materials to improve disposable masks [13,14].

Electrostatic spinning technology for the preparation of nanoscale nonwoven fabrics by a high-voltage electric field force has been widely used for the development of new filter materials [15,16,17,18]. Electrostatic spinning technology can impart higher particle filtration efficiency (PFE) and bacterial filtration efficiency (BFE) and lower breathing resistance to membranes by preparing fiber films with a high specific surface area and high porosity. Nanoscale filters based on PLA have been prepared by this technique and exhibit high filtration efficiency (PM 0.3–99.996%) and a low pressure drop (104 Pa), better than those of commercial N95 filters. Importantly, this filter has long-lasting PM filtration efficiency and natural biodegradability based on PLA [13].

More and more inorganic nanomaterials have been used in electrostatic spinning technology for the development of new filtration materials, such as zinc oxide nanoparticles, carbon nanotubes [19], etc. Nano ZnO are active particles with a particle size in the range of 1–100 nm [20], and this particle size leads to changes in the electronic structure and crystal structure of the ZnO surface. Therefore, nano ZnO possesses properties such as a surface effect [21] and quantum size effect [22] that are not available in the macroscopic state. Interestingly, PLA compounded with nano ZnO can effectively improve the thermal stability and crystallinity of the material [23]. In addition, ZnO nanoparticles are an excellent food-grade antimicrobial agent that can impart antimicrobial effects to air filtration materials while developing films [24,25]. Previous experiments have shown that the surface modification of UHMWPE by grafting with ZnO nanomaterials can result in composites with good antibacterial properties [26]. It has been shown that filters through nanomaterial fibers have a significant impact on efficiency gains in air filtration [27].

In this paper, ZnO-PLLA/PLLA was prepared by the ring-opening polymerization of L-lactide, and ZnO-PLLA/PLLA nanofiber films were fabricated by electrospinning technology. In the synthesis reaction, ZnO nanoparticles provide both an antimicrobial effect for the material and a catalyst for the ring-opening polymerization of DL-lactide. Meanwhile, we intend to utilize the spatial effect of ZnO nanoparticles to enhance the filtration performance of the masks. The effects of the electrospinning process conditions (ZnO-PLLA concentration, ZnO-PLLA/PLLA content, DCM(dichloromethane) to DMF(N,N-dimethylformamide) ratio, and spinning time) on the PEF, airflow resistance (Δp), and quality factor (QF) were explored via orthogonal experiments. The aim is to find a biodegradable, hydrophobic mask filter film material with promising filtration performance through the active exploration of electrospinning technology in the field of air filtration.

## 2. Materials and Methods

### 2.1. Materials

L-lactide (99.9%, PURAC biochem Company, Gorkum, Netherlands), L-polylactide (PLLA, 99.9%, 4032D, NatureWorks LLC, Pennsylvania, NE, USA), nano zinc oxide (ZnO, 99.9%, 30 nm, Klamar Reagent Corporation, Shanghai, China), DCM, and other reagents were purchased from Shanghai Aladdin (Shanghai, China).

### 2.2. Synthesis of ZnO-PLLA Copolymers

**Figure 1:**
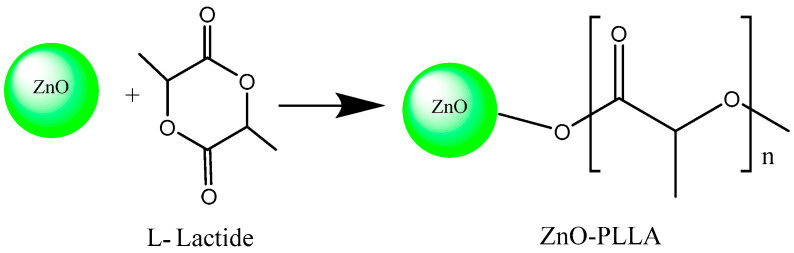
Synthesis route of ZnO-PLLA copolymer.

L-lactide (200 g, 1.4 mol) and ZnO nanoparticles (4 g, 0.05 mol) were added to a 500 mL three-neck flask and nitrogen was displaced three times. The system was evacuated for 2 h while maintaining the internal pressure of −0.08 MPa, and the reaction was carried out at 160 °C for 4 h. The viscosity of the reactant gradually increased from the liquid state and decreased the temperature. DCM was added and the reactants were dissolved and precipitated with anhydrous ethanol. The filtered solid was vacuum-dried at 45 °C for 8 h. Then, 192 g of ZnO-PLLA powder was obtained in 94.1% yield. The synthesis route of ZnO-PLLA is shown in Figure 1.

### 2.3. Synthesis of ZnO-PLLA/PLLA Composites

The above-prepared ZnO-PLLA powder was mixed with 100 g of purchased PLLA by weighing 1, 3, and 5 g, respectively. The mixture was dissolved with 300 mL of DCM and precipitated with 500 mL of anhydrous ethanol. The filtered solid was vacuum-dried at 45 °C for 8 h. According to the ZnO-PLLA content, the as-prepared materials were named 1%ZnO-PLLA/PLLA, 3% ZnO-PLLA/PLLA, and 5%ZnO-PLLA/PLLA.

### 2.4. Structural Characterization of ZnO-PLLA

NMR: 10 mg of ZnO-PLLA was dissolved in 0.5 mL of CDCl_3_ and tested by a nuclear magnetic resonance instrument (NMR, Avance-II 400 MHz, Bruker, Karlsruhe, Germany).

XRD: The structure and crystallization of ZnO-PLLA composites were characterized by an X-ray diffractometer (XRD, D8 ADVANCE, Bruker, Karlsruhe, Germany) with a scan range of 2θ = 5–60°, a scan rate of 5°/min, and a scan step of 0.02°.

### 2.5. Properties of ZnO-PLLA

GPC: The molecular weight and molecular distribution of ZnO-PLLA were measured by gel permeation chromatography (GPC, 2414, Waters, Milford, MA, USA). Polystyrene was used as the standard sample and 1 mL/min of tetrahydrofuran (THF) was used as the eluent.

AAS: 0.2000 g of ZnO-PLLA composite was weighed for microwave digestion. After the digestion solution was fixed to 50 mL, the content of ZnO in the material was measured by an atomic absorption spectrometer (AAS, AA-6300, SHIMADZU, Kyoto, Japan) instrument.

### 2.6. Preparation of ZnO-PLLA/PLLA Electrospun Films

A certain proportion of ZnO-PLLA/PLLA was dissolved in the mixed solution of DCM and DMF. The mixed solution was dissolved uniformly and then ultrasonically defoamed and left to stand, to obtain the spinning solution. Fiber films were prepared by an electrospinning machine (YFSP-T, Yunfan Technology, Tianjin, China). The metal receiver of the electrostatic spinner was wrapped with non-woven fabric. The receiving distance was set to 20 cm, the rotational speed was 50 r/min, the temperature inside the chassis was 25 °C, and the relative humidity was 65%. The spinning solution was loaded into a 5 mL syringe with a metal needle (20-gauge flat tip). Spinning was performed at a voltage of 6–9 kV and a flow rate of 0. 004 mm/s, tuned to a steady Taylor cone jet. The experiments were conducted according to orthogonal experiments with the control factors of ZnO-PLLA content (A), ZnO-PLLA/PLLA content (B), ratio of DCM to DMF (C), and spinning time (D), respectively. The orthogonal experimental factors and level table for these 4 factors are shown in Table 1. After spinning, the films were dried at 45 °C for 8 h by a vacuum drying oven (DZF-6050, Shanghai Precision Instruments Co., Ltd., Shanghai, China). Finally, the fiber film was sandwiched within the non-woven fabric to prepare the mask sample. The thickness of the film was measured with a thickness gauge.

### 2.7. Microscopic Morphology of ZnO-PLLA/PLLA Fiber Films

After the uniform sputtering of a conductive gold layer on the film, the microscopic morphology of the fiber film was observed by a scanning electron microscope (SEM, Nova Nano SEM 450, FEI Company, Portland, OR, USA). The diameter and aperture of the fibers were determined by ImageJ, and the porosity and distribution were calculated.

### 2.8. Particle Filtration Performance of Mask Samples

The particle filtration performance of ZnO-PLLA/PLLA films was tested by a particle protection effect and filtration efficiency tester (PEF, LFY 706, Shandong Institute of Textile Science, Shandong, China) according to Chinese medical surgical mask standard YY0469-2011. The filtration medium was a saline (NaCl) particulate matter aerosol. The test flow rate was controlled at 30 ± 2 L/min. The filtration efficiency particle concentration was 20–30 mg/m^3^ and the median diameter with the salt particulate matter diameter of 0.075 ± 0.02 μm. Each homemade mask specimen was measured 3 times and the average value was taken.

### 2.9. Airflow Resistance of Mask Samples

The respiratory resistance of homemade mask specimens was tested by a medical mask ventilation resistance tester (Δp, LFY 709, Shandong Institute of Textile Science, Shandong, China) according to Chinese medical surgical mask standard YY0469-2011. The specimen permeability was 25 mm. The gas flow rate was 85 ± 2 L/min. Each specimen was measured 3 times.

The quality factor (QF) of the sample filter film was calculated according to Equation (1) to measure the comprehensive performance of the filter film material [28].

QF = −ln(1 − E)/ΔP(1)

where E is the PFE, %; ΔP is the airflow resistance of the specimen film, Pa.

### 2.10. Bacterial Filtration Performance Test of Mask Samples

The bacterial filtration effect of the mask was tested by a mask bacterial filtration efficiency tester (BFE, ZR-1000, JUNRAY, Qingdao, China) according to Chinese medical surgical mask standard YY0469-2011. The experiment used *S. aureus* aerosol particles as a reference. The gas flow rate was 28.3 L/min. The flow rate was 0.180 mL/min. The liquid supply time was 1 min. The sampling time was 2 min, and the colonies were counted on plates after sampling.

## 3. Results and Discussion

### 3.1. Structural Characterization of ZnO-PLLA

**Figure 2:**
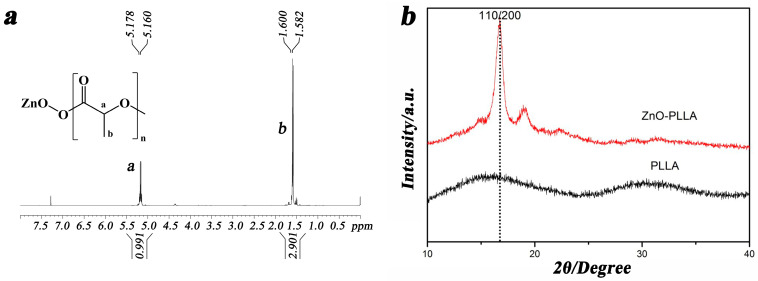
The result of (**a**) H-NMR; (**b**) XPS.

The structure of ZnO-PLLA was characterized by H-NMR. From the NMR spectra in Figure 2a, it is clear that the characteristic peaks of PLLA appear at 1.5 and 5.1 ppm, respectively, which are consistent with the information reported in the literature [29,30]. They are attributed to the proton characteristic peaks of hypomethyl (a) and methyl (b) on the PLLA chain in the chemical structure formula in the figure, respectively.

Figure 2b shows the XRD patterns of PLLA with ZnO-PLLA. As seen in Figure 2b, PLLA has the strongest diffraction peak at 2θ = 16.9°, corresponding to the (110) and (200) crystallographic planes of PLLA, which is the α-crystalline phase. The diffraction peak shapes and positions of ZnO-PLLA are similar to those of pure PLLA, which indicates that the introduction of ZnO has no significant effect on the crystalline structure of PLLA. In addition, the composite films have no characteristic peaks of nano ZnO around 2θ = 10.5°, which indicates that PLLA has been successfully grafted onto the ZnO surface [31,32,33]. Moreover, ZnO reacts well and is uniformly dispersed in the PLLA matrix without agglomerating to produce ZnO stacking structures. The intensity of the diffraction peak of ZnO-PLLA at 16.9° increased significantly; the crystallinity was 40.3%. The new peak near 19.5° may be the result of the uneven molecular weight distribution of ZnO-PLLA and the presence of other by-products reacting with ZnO. The intensity of the diffraction peak increases with the sharpness of the diffraction peak. This indicates that the crystallization ability of ZnO-PLLA has been enhanced.

### 3.2. Content and Molecular Weight of ZnO-PLLA

The GPC results of ZnO-PLLA are shown in Table 2. This shows that the number average molecular weight of ZnO-PLLA is 27755, with a peak time of 15.13 min and a molecular weight distribution of 1.52. There is only a single peak, which indicates that the molecular weight and molecular weight distribution of the polymers are narrow and uniformly dispersed.

### 3.3. Microscopic Morphology of ZnO-PLLA/PLLA Films

**Figure 3:**
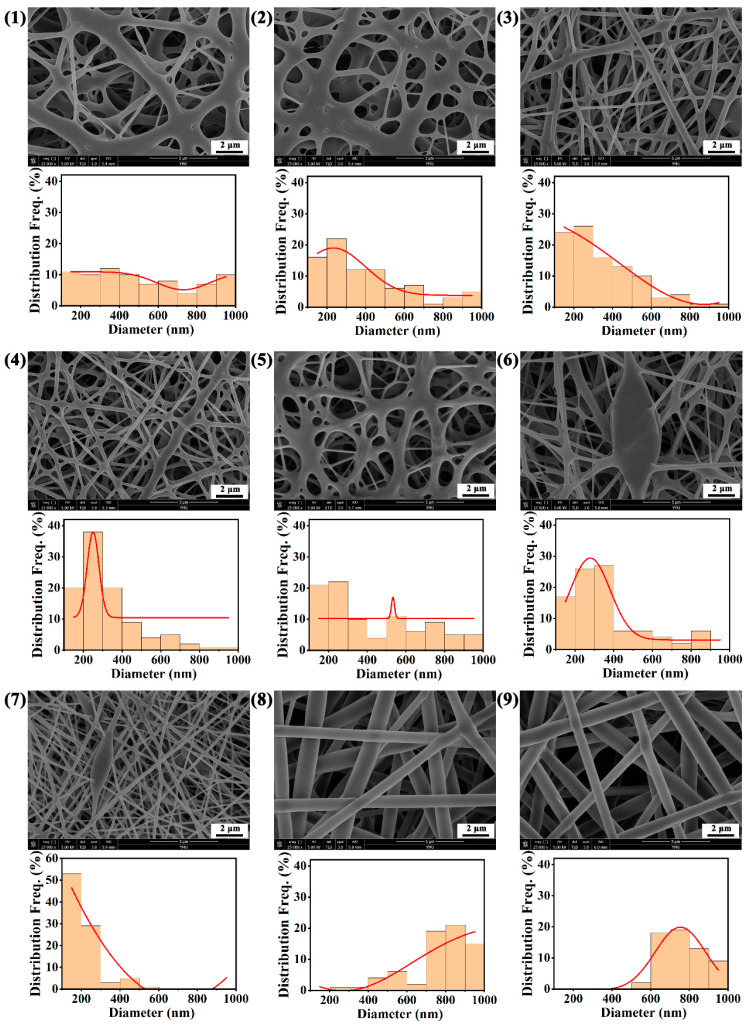
Microscopic morphology and diameter distribution of ZnO-PLLA/PLLA films in groups (**1**–**9**).

Figure 3 shows the microscopic morphology and diameter distribution of ZnO-PLLA/PLLA films. It can be seen from the figure that the morphology of the prepared ZnO-PLLA/PLLA films changes dramatically under different electrostatic spinning conditions. The different composition and content of solute (ZnO-PLLA/PLLA), the ratio of solvent (DCM and DMF), and the time of spinning all lead to different states in the mesh and bead structures of the ZnO-PLLA/PLLA films. The average diameter is distributed from 220 ± 166.41 nm to 928.81 ± 228.11 nm in all nine samples. The smallest fiber diameter and the most uniform distribution are found in group 7.

The porosity of the ZnO-PLLA/PLLA film can be obtained by assessing the results of the micromorphology, which are shown in Table 3. Different microstructures determine differences in porosity. The porosity distribution of ZnO-PLLA/PLLA films ranged from 2.98% to 7.94%.

### 3.4. PFE of ZnO-PLLA/PLLA Films

The PFE of the ZnO-PLLA/PLLA film on particulate matter was tested using NaCl particulate aerosol with a diameter of 0.075 ± 0.02 μm, and the results are shown in Table 3. The PFE distribution was between 70.8 and 98.3, with the three groups of No. 5, 7, and 8 being greater than 95%, which is in accordance with the Chinese medical surgical mask standard YY0469-2011. The results show that the PFE of the fiber film to particulate matter is highly correlated with the structure and distribution of the films. The decrease in fiber diameter may lead to an increase in PFE, which in turn increases the porosity of the ZnO-PLLA/PLLA films. The classical filtration theory consists of five main mechanisms, namely interception, inertial deposition, Brownian diffusion, a gravitational effect, and an electrostatic effect. ZnO-PLLA/PLLA nanofiber filtration films maintain high collection efficiency even at low pressure drops, and effectively capture particles by inertial impingement, interception, and convective Brownian diffusion, even in the absence of electrostatic forces [34]. In addition, the filtration capacity of the film loaded with 5% ZnO-PLLA was significantly better than that of the other components. This is due to the diffusion mechanism of nanoparticle filtration, where the ZnO nanoparticles grafted on PLLA take up part of the gap in the used mask, leading to an increase in filtration [35]. This implies that the grafting of nano ZnO is very effective in enhancing the filtration performance of the ZnO-PLLA/PLLA films.

Among them, No. 0 is a commercially available surgical mask with the PFE of 75.21%, which is significantly lower than all components, proving the superiority of the filter material prepared by electrostatic spinning films.

### 3.5. Airflow Resistance of ZnO-PLLA/PLLA Films

**Figure 4:**
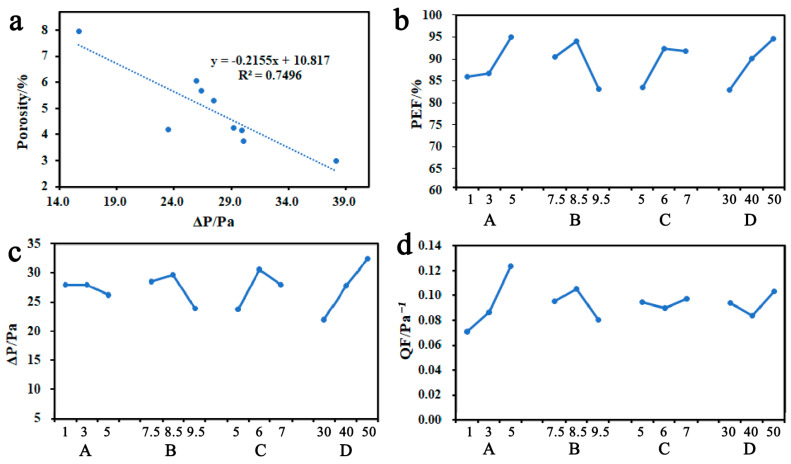
(**a**) The linear relationship between Δp and porosity; the results of the analysis of the orthogonal four factors with PFE (**b**), Δp (**c**), and QF (**d**), respectively.

According to Chinese standard YY0469-2011, the airflow resistance of the mask (Δp, the pressure difference between the two sides of the mask for gas exchange) should be ≤49 Pa. The results of the airflow resistance of ZnO-PLLA/PLLA films are shown in Table 3. The Δp of the nine components ranged from 15.7 Pa to 38.2 Pa, all of which were ≤40 Pa and met the criteria. As shown in Figure 4a, it can be obtained that the airflow resistance is linearly correlated with the porosity, and the correlation coefficient R2 is 0.7496. Therefore, the porosity is the main factor affecting the airflow resistance. From the electron microscopy results in Figure 4b, it can be seen that the No. 6 group of beads has an obvious structure and larger beads. The lowest respiratory resistance of the film is 15.7 Pa at this time. The beaded structure is typical of an elliptical protruding morphology along the fibers, which is a state of slip between polymer chains due to the fragmentation of polymer jets at low concentrations, caused by Rayleigh instability [36,37]. It is shown that a large and uniform bead structure can significantly reduce the airflow resistance of electrostatic spinning films, which is consistent with the experimental results [38].

Only the Δp of the filter material of No. 5 is higher than that of the commercially available mask of No. 0. The gas resistance of all other components is smaller than that of No. 0, which proves the low breathing resistance of the electrostatic spun silk film.

The quality factor (QF) is actually the ratio of the filtration efficiency to the filtration pressure drop. The larger the QF, the better the filtration performance [39]. Compared with conventional fiber filtration materials, nanofiber filtration films have higher filtration efficiency and a lower filtration pressure drop [40] with a greater QF. The QF results of ZnO-PLLA/PLLA films are shown in Table 3. The QF distribution ranges from 0.0641 to 0.1403, all higher than 0.0628 for No. 0, and the highest quality factor was found for nanofiber films in group No. 7.

### 3.6. Thickness and Aperture

The results of the thickness and pore size of the fiber films are shown in Table 4. The thickness distribution of the films ranged from 10.00 to 27.67 µm. Comparison of the spinning times of the corresponding components shows that the correlation between thickness and spinning time is high. Overall, there is a tendency for PFE to increase as the thickness increases, but the performance of PFE is more correlated with the microstructure of the fiber (pore size, alignment, porosity, etc.).

**Figure 5:**
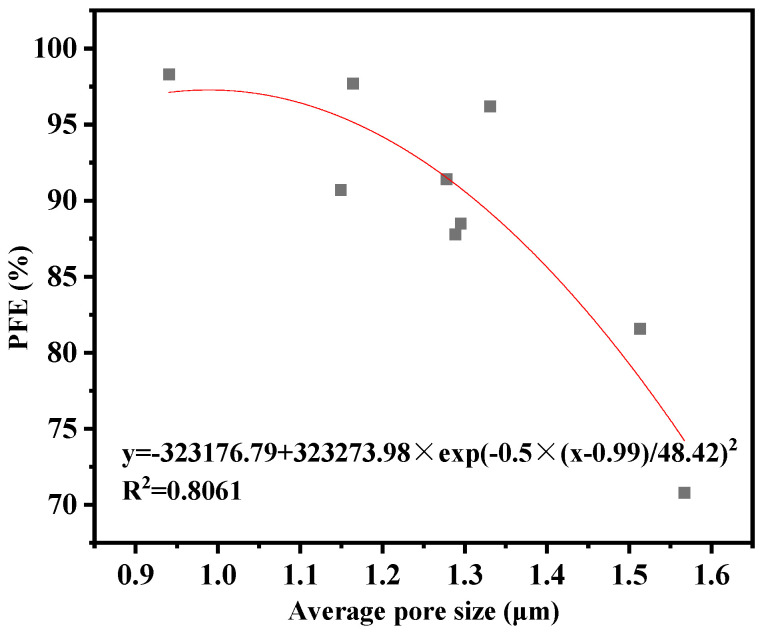
Correlation of pore size and PEF.

The average pore size distribution of the fibers is between 0.94 and 1.87 microns. The correlation between pore size and PFE is shown in Figure 5, from which it can be seen that the correlation between pore size and PFE is strong, with a correlation coefficient of 0.8061. In addition, PFE is also related to the degree of uniformity in the distribution of pore size.

### 3.7. Results of L_9_ (4^3^) Orthogonal Test

In this study, the optimized process parameters of the ZnO-PLLA /PLLA nanometer antibacterial fiber film were obtained by the L_9_ (4^3^) orthogonal test. The test indexes were the PFE of the fiber film, the airflow resistance, and the QF. The experimental protocols and results of the preparation of electrostatic spinning films based on orthogonal arrays are shown in Table 3. Only the effects of A, B, C, and D on the experimental results were considered during the experiment, and the interactions between the factors were not considered.

The results of the analysis of the orthogonal four factors with PEF, Δp, and QF are shown in Figure 4b–d. Based on the extreme difference R, it can be judged that the four factors have the same pattern of influence on PFE and Δp: spinning time (D) > ZnO-PLLA/PLLA content (B) > ratio of DCM to DMF (C) > ZnO-PLLA content (A). The data show that an increase in spinning time significantly increases the PFE and Δp values. However, the smaller the Δp, the better, and an excessive increase in Δp can lead to a decrease in the breathability of the mask. The introduction of nano ZnO is beneficial to the PFE enhancement, but will only slightly increase the airflow resistance.

Interestingly, the effects of the four factors on QF were as follows: ZnO-PLLA content (A) > ZnO-PLLA/PLLA content (B) > spinning time (D) > ratio of DCM to DMF (C). This is in agreement with the findings of PFE and Δp, proving that ZnO nanoparticles in ZnO-PLLA are significant in enhancing the quality of the masks. Among them, the spinning time mainly affects the thickness of the fiber film. The ZnO-PLLA/PLLA content and the ratio of DCM to DMF affect the morphology of the fiber film by changing the viscosity and electrical conductivity of the solution. ZnO-PLLA can enhance the filtration capacity of the fiber. Based on the results of the orthogonal experiments, it can be obtained that if we wish to further improve the quality of the masks, we can use scheme A_3_B_2_C_3_D_3_ for the experiments, i.e., ZnO-PLLA/PLLA with a mass fraction of 9.5%, where the ZnO loading is 5% and the DCM/DMF mass ratio is 7:1 for 50 min.

### 3.8. Bacterial Filtration Capacity of ZnO-PLLA/PLLA Films

The three components with the highest particle filtration efficiency (No. 5, No. 7, and No. 8) were selected for bacterial filtration tests, and the conclusions drawn are shown in Table 3. As can be seen from the table, the bacterial filtration efficiency of all three samples is greater than 96%, and all of them meet the Chinese medical surgical mask standard YY0469-2011. The efficiency of sample No. 7 is the highest, reaching 98.42%. Meanwhile, the results of bacterial filtration are highly correlated with the results of particulate filtration. This proves the potential of ZnO-PLLA/PLLA films in the field of medical mask cartridge materials.

## 4. Conclusions

In this paper, ZnO-PLLA/PLLA was prepared by the polymerization of ZnO nanoparticles with L-lactide, and ZnO-PLLA/PLLA fiber films for air filtration were prepared via the electrostatic spinning technique. The nano ZnO itself is a good catalyst that can greatly facilitate the reaction. Meanwhile, the surface of ZnO contains a large number of hydroxyl groups, which can further trigger the ring-opening polymerization of L-lactide. The results show that this method can graft ZnO onto the PLLA surface directly and quickly by a one-step reaction, which changes the chemical structure of the ZnO surface layer. This method provides a new idea and method to solve the problem of phase separation between inorganic nanomaterials and organic polymer materials.

In addition, the process parameters of ZnO-PLLA /PLLA nanofiber films were investigated and optimized by L_9_ (43) orthogonal experiments in this study. The particle filtration efficiency (PFE), airflow resistance (Δp), and quality factor (QF) of the films were investigated. The effects of the four factors on the QF in descending order are ZnO-PLLA content (A), ZnO-PLLA/PLLA content (B), spinning time (D), and the ratio of DCM to DMF (C). Among them, the introduction of ZnO-PLLA is the most important factor to enhance the QF of the filter material. The optimal fraction obtained experimentally was sample number seven, where the QF was 0.1403 Pa^−1^, the PFE was 98.3%, the BFE was 98.42%, and the Δp was 29.2 Pa. In summary, ZnO-PLLA/PLLA nanofiber films have outstanding development potential and advantages in the field of air filtration.

## Figures and Tables

**Table 1:** Orthogonal experimental design of electrostatic spinning film.

Level	Factor			
	ZnO-PLLA Content/%	ZnO-PLLA/PLLA Solute Content/%	Ratio of DCM to DMF	Spinning Time/min
1	1	7.50	5	30
2	3	8.50	6	40
3	5	9.50	7	50

**Table 2:** GPC results of ZnO-PLLA.

Retention Time	Mn	Mw	Mp	Polydispersity
15.13	27,755 ± 48	36,808 ± 68	23,426 ± 38	1.52 ± 0.01

**Table 3:** Results of L_9_ (4^3^) orthogonal experiments, filtration efficiency, respiratory resistance, porosity, mean diameter, and bacterial filtration.

Test No.	A	B	C	D	PFE/%	Δp/Pa	QF/Pa^−1^	Porosity/%	Mean Diameter/nm	BFE/%
0	–	–	–	–	75.21	34.18	0.0628	–	–	–
1	1	1	1	1	81.6	26.4	0.0641	5.67	669.91 ± 418.71	–
2	1	2	2	2	88.5	27.4	0.0787	5.28	571.60 ± 506.01	–
3	1	3	3	3	87.8	30.0	0.0703	4.16	367.05 ± 234.28	–
4	2	1	3	2	91.4	30.1	0.0816	3.74	321.51 ± 164.23	–
5	2	2	2	3	97.7	38.2	0.0988	2.98	472.69 ± 319.17	97.52
6	2	3	1	1	70.8	15.7	0.0786	7.94	478.36 ± 577.08	–
7	3	1	1	3	98.3	29.2	0.1403	4.25	220.38 ± 166.41	98.42
8	3	2	3	1	96.2	23.5	0.1390	4.18	914.91 ± 262.93	96.11
9	3	3	2	2	90.7	25.9	0.0916	6.07	928.81 ± 228.11	–
			A		B		C		D	
PFE/%										
k_1_			86.0		90.4		83.6		82.9	
k_2_			86.6		94.1		92.3		90.2	
k_3_			95.1		83.1		91.8		94.6	
R			8.4		11.0		8.7		11.7	
Δp/Pa										
k_1_			27.9		28.6		23.8		21.9	
k_2_			28.0		29.7		30.5		27.8	
k_3_			26.2		23.9		27.9		32.4	
R			1.8		5.9		6.8		10.6	
QF/Pa^−1^										
k_1_			0.0710		0.0953		0.0943		0.0939	
k_2_			0.0863		0.1055		0.0897		0.0840	
k_3_			0.1236		0.0802		0.0970		0.1031	
R			0.0526		0.0253		0.0073		0.0192	

**Table 4:** Thickness and aperture of fiber film.

	1	2	3	4	5	6	7	8	9
Thickness/μm	14.67 ± 0.47	18.00 ± 0.00	23.33 ± 0.47	17.33 ± 0.94	21.33 ± 2.36	10.00 ± 0.82	27.67 ± 0.47	11.00 ± 0.82	15.67 ± 0.47
Aperture/μm	1.51 ± 1.08	1.30 ± 0.60	1.29 ± 0.80	1.28 ± 0.64	1.16 ± 0.83	1.57 ± 1.13	0.94 ± 0.93	1.33 ± 1.20	1.87 ± 0.99

## Data Availability

No new data were created.

## References

[1] Dharmaraj S., Ashokkumar V., Hariharan S., Manibharathi A., Show P.L., Chong C.T., Ngamcharussrivichai C. (2021). The COVID-19 Pandemic Face Mask Waste: A Blooming Threat to the Marine Environment. Chemosphere.

[2] Deng W., Sun Y., Yao X., Subramanian K., Ling C., Wang H., Chopra S.S., Xu B.B., Wang J., Chen J. (2022). Masks for COVID-19. Adv. Sci..

[3] Gupta D.K., Vishwakarma A., Singh A. (2023). Release of Microplastics from Disposable Face Mask in Tropical Climate. Reg. Stud. Mar. Sci..

[4] Miah M.J., Pei J., Kim H., Sharma R., Jang J.G., Ahn J. (2023). Property Assessment of an Eco-Friendly Mortar Reinforced with Recycled Mask Fiber Derived from COVID-19 Single-Use Face Masks. J. Build. Eng..

[5] Zou Q., Gai Y., Cai Y., Gai X., Xiong S., Wei N., Jiang M., Chen L., Liu Y., Gai J. (2022). Eco-Friendly Chitosan@silver/Plant Fiber Membranes for Masks with Thermal Comfortability and Self-Sterilization. Cellulose.

[6] Stanislas T.T., Bilba K., de Oliveira Santos R.P., Onésippe-Potiron C., Savastano Junior H., Arsène M.-A. (2022). Nanocellulose-Based Membrane as a Potential Material for High Performance Biodegradable Aerosol Respirators for SARS-CoV-2 Prevention: A Review. Cellulose.

[7] Wang L., Peng Y., Xu Y., Zhang J., Liu C., Tang X., Lu Y., Sun H. (2022). Earthworms’ Degradable Bioplastic Diet of Polylactic Acid: Easy to Break down and Slow to Excrete. Environ. Sci. Technol..

[8] Wang X., Chen K., Liu Y., He R., Wang Q. (2023). Preparation and Application of Biodegradable and Superhydrophobic Polylactic Acid/Carnauba Wax Coating. Prog. Org. Coat..

[9] Wang X., Pan Y., Liu X., Liu H., Li N., Liu C., Schubert D.W., Shen C. (2019). Facile Fabrication of Superhydrophobic and Eco-Friendly Poly(Lactic Acid) Foam for Oil–Water Separation via Skin Peeling. ACS Appl. Mater. Interfaces.

[10] Wu Y., Hao X., Lin F., Wang S., Chen L., Lin X., Gan D., Fan S., Song L., Liu Y. (2022). Developing a Cerium Lactate Antibacterial Nucleating Agent for Multifunctional Polylactic Acid Packaging Film. Int. J. Biol. Macromol..

[11] Wei Y., Bai J., Zhao H., Wang R., Li H. (2022). Study on the Extrusion Molding Process of Polylactic Acid Micro Tubes for Biodegradable Vascular Stents. Polymers.

[12] Huang J., Xie G., Wei Q., Su Y., Xu X., Jiang Y. (2023). Degradable MXene-Doped Polylactic Acid Textiles for Wearable Biomonitoring. ACS Appl. Mater. Interfaces.

[13] Wang L., Gao Y., Xiong J., Shao W., Cui C., Sun N., Zhang Y., Chang S., Han P., Liu F. (2022). Biodegradable and High-Performance Multiscale Structured Nanofiber Membrane as Mask Filter Media via Poly(Lactic Acid) Electrospinning. J. Colloid Interface Sci..

[14] Zalewski E.F., Geist J. (1980). Silicon Photodiode Absolute Spectral Response Self-Calibration. Appl. Opt..

[15] Zhang Q., Li Q., Young T.M., Harper D.P., Wang S. (2019). A Novel Method for Fabricating an Electrospun Polyvinyl Alcohol/Cellulose Nanocrystals Composite Nanofibrous Filter with Low Air Resistance for High-Efficiency Filtration of Particulate Matter. ACS Sustain. Chem. Eng..

[16] Zhu M., Hua D., Pan H., Wang F., Manshian B., Soenen S.J., Xiong R., Huang C. (2018). Green Electrospun and Crosslinked Poly(Vinyl Alcohol)/Poly(Acrylic Acid) Composite Membranes for Antibacterial Effective Air Filtration. J. Colloid Interface Sci..

[17] Deng Y., Lu T., Zhang X., Zeng Z., Tao R., Qu Q., Zhang Y., Zhu M., Xiong R., Huang C. (2022). Multi-Hierarchical Nanofiber Membrane with Typical Curved-Ribbon Structure Fabricated by Green Electrospinning for Efficient, Breathable and Sustainable Air Filtration. J. Membr. Sci..

[18] Lyu C., Zhao P., Xie J., Dong S., Liu J., Rao C., Fu J. (2021). Electrospinning of Nanofibrous Membrane and Its Applications in Air Filtration: A Review. Nanomaterials.

[19] Duong H.M., Tran T.Q., Kopp R., Myint S.M., Peng L. (2019). Direct Spinning of Horizontally Aligned Carbon Nanotube Fibers and Films from the Floating Catalyst Method. Nanotube Superfiber Materials.

[20] Dutta G., Sugumaran A. (2021). Bioengineered Zinc Oxide Nanoparticles: Chemical, Green, Biological Fabrication Methods and Its Potential Biomedical Applications. J. Drug Deliv. Sci. Technol..

[21] Combe N., Chassaing P.-M., Demangeot F. (2009). Surface Effects in Zinc Oxide Nanoparticles. Phys. Rev. B.

[22] Li N., Gao Y., Hou L., Gao F. (2011). DNA-Based Toolkit for Directed Synthesis of Zinc Oxide Nanoparticle Chains and Understanding the Quantum Size Effects in ZnO Nanocrystals. J. Phys. Chem. C.

[23] Bu Y., Chen Z., Li W., Hou B. (2013). Highly Efficient Photocatalytic Performance of Graphene–ZnO Quasi-Shell–Core Composite Material. ACS Appl. Mater. Interfaces.

[24] Huang X., Zhou X., Dai Q., Qin Z. (2021). Antibacterial, Antioxidation, UV-Blocking, and Biodegradable Soy Protein Isolate Food Packaging Film with Mangosteen Peel Extract and ZnO Nanoparticles. Nanomaterials.

[25] Luo Y., Zhai F., Zhang Y., Chen Z., Ding M., Qin D., Yang J., Feng G., Li L. (2021). A Superfine Glass Fibre Air Filter with Rapid Response to Photocatalytic Antibacterial Properties under Visible Light by Loading RGO/ZnO. R. Soc. Open Sci..

[26] Benkocká M., Lupínková S., Knapová T., Kolářová K., Matoušek J., Slepička P., Švorčík V., Kolská Z. (2019). Antimicrobial and Photophysical Properties of Chemically Grafted Ultra-High-Molecular-Weight Polyethylene. Mater. Sci. Eng. C.

[27] Kim J., Chan Hong S., Bae G.N., Jung J.H. (2017). Electrospun Magnetic Nanoparticle-Decorated Nanofiber Filter and Its Applications to High-Efficiency Air Filtration. Environ. Sci. Technol..

[28] Thavasi V., Singh G., Ramakrishna S. (2008). Electrospun Nanofibers in Energy and Environmental Applications. Energy Environ. Sci..

[29] Deng X.M., Yuan M.L., Xiong C.D., Li X.H. (1999). Polymerization of Lactides and Lactones. II. Ring-Opening Polymerization of ε-Caprolactone and DL-Lactide by Organoacid Rare Earth Compounds. J. Appl. Polym. Sci..

[30] Deng X., Yuan M., Li X., Xiong C. (2000). Polymerization of Lactides and Lactones VII. Ring-Opening Polymerization of Lactide by Rare Earth Phenyl Compounds. Eur. Polym. J..

[31] Zhou X., Shi T., Zhou H. (2012). Hydrothermal Preparation of ZnO-Reduced Graphene Oxide Hybrid with High Performance in Photocatalytic Degradation. Appl. Surf. Sci..

[32] Yang Y., Ren L., Zhang C., Huang S., Liu T. (2011). Facile Fabrication of Functionalized Graphene Sheets (FGS)/ZnO Nanocomposites with Photocatalytic Property. ACS Appl. Mater. Interfaces.

[33] Zhu J., Zeng G., Nie F., Xu X., Chen S., Han Q., Wang X. (2010). Decorating Graphene Oxide with CuO Nanoparticles in a Water–Isopropanol System. Nanoscale.

[34] Wang C.-S. (2001). Electrostatic Forces in Fibrous Filters—A Review. Powder Technol..

[35] Bansal P., Batra R., Yadav R., Purwar R. (2022). Electrospun Polyacrylonitrile Nanofibrous Membranes Supported with Montmorillonite for Efficient PM2.5 Filtration and Adsorption of Cu (II) Ions. J. Appl. Polym. Sci..

[36] Shenoy S.L., Bates W.D., Frisch H.L., Wnek G.E. (2005). Role of Chain Entanglements on FIber Formation during Electrospinning of Polymer Solutions: Good Solvent, Non-Specific Polymer–Polymer Interaction Limit. Polymer..

[37] Hohman M.M., Shin M., Rutledge G., Brenner M.P. (2001). Electrospinning and Electrically Forced Jets. I. Stability Theory. Phys. Fluids.

[38] Kadam V., Kyratzis I.L., Truong Y.B., Schutz J., Wang L., Padhye R. (2019). Electrospun Bilayer Nanomembrane with Hierarchical Placement of Bead-on-String and Fibers for Low Resistance Respiratory Air Filtration. Sep. Purif. Technol..

[39] Munir M.M., Adrian M., Burhanuddin M., Iskandar F. (2022). Fabrication and Structure Optimization of Expanded Polystyrene (EPS) Waste Fiber for High-Performance Air Filtration. Powder Technol..

[40] Wu J., Akampumuza O., Liu P., Quan Z., Zhang H., Qin X., Wang R., Yu J. (2020). 3D Structure Design and Simulation for Efficient Particles Capture: The Influence of Nanofiber Diameter and Distribution. Mater. Today Commun..

